# *BRCA2* Mutations and Triple-Negative Breast Cancer

**DOI:** 10.1371/journal.pone.0038361

**Published:** 2012-05-30

**Authors:** Peter Meyer, Katharina Landgraf, Bernhard Högel, Wolfgang Eiermann, Beyhan Ataseven

**Affiliations:** 1 Institute of Medical Genetics, University Hospital, Rostock, Germany; 2 Institute of Pathology, Red Cross Hospital, Munich, Germany; 3 Frauenklinik vom Roten Kreuz, Munich, Germany; Ohio State University Medical Center, United States of America

## Abstract

Recently, *BRCA1* germline mutations were found in a high proportion (14–34%) of patients with triple-negative breast cancer (TNBC). *BRCA2* was either not analyzed or showed much lower mutation frequencies. Therefore, we screened a group of TNBC patients (n = 30) of white European descent for mutations in *BRCA2* as well as in *BRCA1*. Cases were unselected for age of disease-onset (median age at breast cancer diagnosis was 58 years, ranging from 37 to 74 years), family history of cancer and *BRCA1* and *BRCA2* mutation status. Half of the patients (15/30) showed a family history of breast and/or ovarian cancer. A high frequency of deleterious germline mutations was observed in *BRCA2* (5/30; 16.7%), and only one case showed a *BRCA1* mutation (3.3%). Although the study group was small, these results point to *BRCA2* mutations being important in TNBC.

## Introduction

Recently, triple-negative breast cancer (TNBC) has been classified as a breast cancer subgroup that is negative for estrogen and progesterone receptors (ER/PR) and receptor 2 of human epidermal growth factor (HER2). TNBC accounts for approximately 15% of all breast cancer cases and seems to be closely related to basal-like breast cancer, which has an expression profile similar to that of normal basal-myoepithelial breast tissue. Approximately 80% of TNBC cases show a basal-like phenotype, and the majority of basal-like breast cancers can be classified as TNBC. Close to 75% of *BRCA1*-associated breast cancers is either TNBC, basal-like breast cancer or both. TNBC patients have a relatively poor outcome and cannot be treated with endocrine therapy or therapies targeted to HER2 due to the lack of related receptors [1].

Recent reports have focussed on *BRCA1* germline mutations in TNBC cases. In a Canadian TNBC cohort (n = 54) with an age of onset before 41 years and no familial breast cancer aggregation, five cases with mutations of *BRCA1* (9%) and only one case with a mutation of *BRCA2* (2%) were detected. The entire coding sequence of *BRCA1* and the large exons 10 and 11 of *BRCA2* were analyzed [2]. Of 73 TNBC cases from the United Kingdom (UK) studied, 16 (22%) had *BRCA1* mutations and none had *BRCA2* mutations when fully screened in both genes. Forty-three of these cases were from patients younger than 41 years of age at diagnosis and with no familial aggregation of breast cancer. An additional 30 cases were unselected for family history of breast cancer because they were younger than 31 years old at onset of the disease [3]. In 77 TNBC cases from the United States that were not selected for age or familial breast cancer occurrence, the mutation frequencies were 15.6% for *BRCA1* (n = 12) and 3.9% for *BRCA2* (n = 3). The entire coding sequence and the exon-intron boundaries were screened in both genes [4]. Finally, a cohort of 64 Ashkenazi Jewish TNBC patients unselected for age of onset and familial aggregation of breast cancer was screened for mutations in *BRCA1* and *BRCA2*. In all Ashkenazi Jewish breast cancer cases, approximately 10% of patients carry one of three different founder mutations in *BRCA1* and *BRCA2*. In the investigated Ashkenazi Jewish TNBC cohort (n = 64), 19 *BRCA1* (29.7%) and 6 *BRCA2* (9.4%) mutations could be detected when screening for the three founder mutations [5]. Given that the recent reports on TNBC cases describe either no or low frequencies of *BRCA2* mutations [3], [4], or refer to very special inbred populations with high founder mutation frequencies [5], or report on incomplete *BRCA2* mutation screening approaches [2], we aimed to investigate the *BRCA2* mutation frequency in a TNBC cohort of a Southern German population unselected for age of onset and familial aggregation of cancer. If there was a relevant mutation rate in such cases, TNBC would meet the clinical criteria for mutation screening in *BRCA2* and should be added to the current guidelines.

## Methods

### Ethics statement

All selected patients underwent genetic counselling and gave their written informed consent for genetic testing. The study protocol was approved by the Ethics Committee of the State of Salzburg (Austria).

### Study population

In a monocentric approach executed in Munich, Germany, newly diagnosed cases of individuals with TNBC diagnosed between 2005 and 2010 were selected from the Pathology Unit. Histological samples were classified as TNBC when the following criteria were met: less than 1% of cells demonstrated nuclear staining for estrogen and progesterone receptors, and immunohistochemical staining for HER2 showing a 0, 1-fold, or a 2-fold positive score and a FISH ratio (HER2 gene signals to chromosome 17 signals) of less than 1.8 according to the relevant ASCO and CAP guidelines [6]. No further selection criteria were applied. For immunohistochemistry (IHC), we used IgG_1_ mouse antibodies for estrogen (clone 6F11) and for progesterone receptors (clone 16; both antibodies were purchased from DCS Innovative Diagnostik-Systeme, Dr. Christian Sartori GmbH & Co. KG, Hamburg, Germany). For HER2-IHC we used polyclonal rabbit antibodies from clone A0485 (Hercep™-Test; Dako Deutschland GmbH, Hamburg, Germany).

### Genotyping

DNA extraction from whole blood samples (EDTA) was performed according to standard protocols. To amplify exons and exon-intron boundaries of *BRCA1* and *BRCA2*, we used primer pairs and polymerase chain reaction (PCR) protocols published elsewhere [7]. PCR products were sequenced using BigDye® Terminator Cycle Sequencing Kits and 3730xl DNA Analyzers with 96 capillary arrays according to the manufacturer's protocols (Applied Biosystems, Foster City, CA 94404 USA). To exclude deletions and duplications of one or more exons, we performed Multiplex Ligation-dependent Probe Amplification (MLPA) of both genes according to the company's technical protocols (MRC-Holland, Amsterdam, the Netherlands). Mutations and variants were classified according to the kConFab classification scheme [8].

### Clinical criteria for mutation screening in *BRCA1* and *BRCA2*

In Germany, patients with a 10% mutation probability are eligible for mutation screening in *BRCA1* and *BRCA2*. The following clinical criteria were evaluated in approximately 1,000 unrelated cases in a German-wide study:

- Two women with breast cancer, with one of them ≤50 years, or three women with breast cancer, independent of age.
- One woman with breast and one woman with ovarian cancer, independent of age, or one woman with breast and ovarian cancer, independent of age, or two women with ovarian cancers, independent of age.
- One male breast cancer and one woman with breast or ovarian cancer.
- One woman with breast cancer ≤35 years.
- One woman with bilateral breast cancer or two primary breast cancers, with first tumour diagnosed before ≤50 years [9], [10].

## Results

Characteristics of investigated patients are presented in Table 1. Six out of 30 TNBC patients (20%) carried a deleterious mutation – one (1/30; 3.3%) in *BRCA1* and five (5/30; 16.7%) in *BRCA2*. We detected four known deleterious frame-shift mutations (*BRCA1*: c.5266dupC (old nomenclature is “5382insC”) which is one of the most common deleterious *BRCA1* mutations [11]; *BRCA2*: c.1029delA [12], p.S1882X [13], c.6444dupT accession number 3790 in BIC database [http://research.nhgri.nih.gov]) and one unknown splice-site mutation (*BRCA2*: c.476-1G>A) with a clear predictive deleterious effect. One missense mutation in *BRCA2* (p.W2626C) was classified as “predictive deleterious” in prior literature [13].

**Table 1:** Characteristics of TNBC Cases.

DNA	BRCA1 Category	BRCA1 Mutation	BRCA2 Category	BRCA2 Mutation	Age at Diagnosis	Second Tumor (Age)	Family History of Cancer (Age)	Criteria for Mutation Screening*
**a) Mutation Carriers**								
1110	LCS	p.R496H	**PM**; RP (TNBC)	**c.1029delA**; p.T1915M	69	0	6 x BC ms	yes
1129	-	-	**PrPM**	**p.W2626C**	61	0	4x lung cancer ms, grandmother ms BC (61), 4 add. cancers ms	no
1153	**PM**	c.**5266dupC**	-	-	43	BC (48)	mother BC (60)	yes
1156	-	-	**PM**	**p.S1882X**	37	0	0	no
1186	-	-	**PM**	**c.476-1G>A**	56	0	sister BC (52), mother OvCa (63)	yes
1245	-	-	**PM**	**c.6444dupT**	38	BC (43)	mother OvCa (60)	yes
				**Median**	**50**			**4/6 (67%)**
**b) Remaining TNBC Patients**								
1102	-	-	LCS	p.A2951T	38	0	0	no
1106	-	-	RP (TNBC)	p.T1915M	48	BC (56)	mother BC (45)	yes
1108	-	-	-	-	71	0	0	no
1109	-	-	-	-	63	skin	sister BC (58)	no
1111	-	-	-	-	64	0	aunt ms BC (68), aunt ms BC (50)	yes
1115	LCS	p.M1652I	-	-	59	0	0	no
1117	-	-	-	-	57	basalioma (56)	0	no
1118	-	-	-	-	51	uterus (20; cis)	sister BC (50)	yes
1130	-	-	-	-	70	0	mother BC (41)	yes
1132	-	-	-	-	43	0	aunt fs BC (42+50)	yes
1135	-	-	-	-	48	0	0	no
1139	LCS	p.M1652I	-	-	43	BC (57), basalioma (58)	0	yes
1141	-	-	-	-	74	0	0	no
1145	-	-	US	c.68-7T>A	57	0	0	no
1178		-	RP (TNBC)	p.T1915M	70	0	aunt ms OvCa	yes
1180	-		US	p.F1524V	49	0	0	no
1182	-	-	-	-	63	0	3 x BC ms, 2x lung cancer ms	yes
1201	-	-	RP (TNBC)	p.T1915M	63	0	sister of grandmother ms BC	no
1217	-	-	-	-	50	0	0	no
1219	-	-	-	-	70	0	cousin ms BC (40)	yes
1232	LCS	p.M1652I	-	-	63	0	niece bilateral BC (32), aunt ms BC (42)	yes
1244	-	-	-	-	66	0	aunt fs BC	no
1252	LCS	p.M1652I	-	-	68	0	sister BC (66), grandmother fs BC (50)	yes
1259	-	-	-	-	56	0	0	no
				**Median**	**61**			**11/24 (46%)**
							**Total**	**15/30 (50%)**

**Abbreviations:** TNBC: triple negative breast cancer; BC/OvCa: breast cancer, ovarian cancer; LCS: low clinical significance; PM: pathogenic mutation; PrPM: predictive PM; RP: risk conferring polymorphism; US: unknown significance; ms: maternal; fs: paternal. *Criteria for mutation screening: Details are described in the Methods section.

In four cases, we detected c.4956G>A in *BRCA1* leading to an exchange of methionine for isoleucine at amino acid position 1,652 of the protein (p.M1652I; 4/30; 13.3%). Variant c.5744C>T causes a point mutation of threonine to methionine at amino acid position 1,915 of *BRCA2* and was detected in four cases (p.T1915M; 4/30; 13.3%). Within mutation carriers, two out of six cases (33.3%) did not meet the German clinical criteria for *BRCA1* and *BRCA2* testing. From all TNBC cases, 15 out of 30 (50%) showed an early age of onset or a familial aggregation which are German clinical criteria for mutational screening of *BRCA1* and *BRCA2*. The median age at diagnosis of the total study group was 58 years (n = 30), for mutations carriers 50 years (n = 6), and for the remaining patients (n = 24) not showing deleterious mutations 61 years.

## Discussion

The aim of this study was to investigate the role of *BRCA2* germline mutations in triple-negative breast cancer (TNBC). We detected 6 deleterious *BRCA1* and *BRCA2* mutations in 30 TNBC patients (20%). The total mutation frequency for both genes is comparable with those described in recent studies [5], [3], [4], [2]. In contrast, we found a high frequency of *BRCA2* germline mutations in our TNBC study group: four cases with previously published deleterious variants, and one splice-site mutation which was not published before (16.7%). The low rate of *BRCA2* mutations found in a Canadian sample (1/54 = 2%) was probably the result of assay design given that only exons 10 and 11 of *BRCA2* were tested. Additionally, only TNBC cases with an age of disease onset of less than 41 years were selected for the study [2]. Our patient group was not selected for age of disease onset; the average age was 57 years, with a median age of 58 years. This age difference between the two study groups could also explain the inconsistent frequencies of *BRCA2* mutations. No *BRCA2* mutations were detected in 73 TNBC cases from the UK. *BRCA1* and *BRCA2* genes were fully screened, but patients were diagnosed with TNBC before age 41 (n = 43) and before age 30 (n = 30). Cases were selected for the UK study only if no familial aggregation was reported [3]. In contrast, our TNBC group was not selected for familial tumour aggregation or age of onset; these differences in inclusion criteria may explain the different *BRCA2* mutation frequencies. Interestingly, a similar study of 77 TNBC cases from the United States where cases were unselected for age of onset (average was 51 years) revealed a much lower *BRCA2* mutation rate (3.9% vs. 16.7% in our study) [4].

Recently, data of a multi-national approach containing approximately 7,000 *BRCA1* and *BRCA2* mutation carriers (*BRCA1*: n = 4,325; *BRCA2*: n = 2,568) showed that the proportion of TNBC increased with age at diagnosis in *BRCA2* mutation carriers, while it decreased in *BRCA1* mutation carriers [14]. These findings are in accordance with our results since our patient group tended to be older than those reporting lower *BRCA2* and higher *BRCA1* mutations rates in TNBC [5], [3], [4], [2].

In four cases, we detected c.4956G>A in *BRCA1* (p.M1652I; 4/30; 13.3%). Analysis with protein prediction software showed some minor effect [15], so we decided to classify this variant as reaching, at most, “low clinical significance” (LCS; table 1). Variant c.5744C>T was detected in four cases (p.T1915M; 4/30; 13.3%). Methionine at amino acid position 1,915 of *BRCA2* (rs4987117) was less frequent (OR 0.39, 95% CI 0.19 – 0.82, p = 0.013) in estrogen receptor-positive patients of a study cohort of 117 sporadic breast cancer cases from Poland [16]. We therefore classified this variant as “risk conferring polymorphism” (table 1). The two variants (c.4956G>A in *BRCA1* and c.5744C>T in *BRCA2*) could therefore be investigated in much larger association studies to evaluate their impact in TNBC tumorigenesis.

We identified deleterious *BRCA2* mutations in two patients not meeting the current German clinical criteria for *BRCA1* and *BRCA2* mutation screening. In our cohort of TNBC patients unselected for age at diagnosis and cancer family history, 15 cases (15/30; 50%) did not meet those criteria. The mutation rate in this subgroup was 13% (2/15). The main requirement for the German clinical criteria for *BRCA1* and *BRCA2* mutation testing was defined as being at least a 10% a priori probability to carry either a *BRCA1* or *BRCA2* mutation [9], [10]. If larger studies confirm our findings, TNBC therefore may be included within clinical criteria for *BRCA1* and *BRCA2* mutation screening, and additional cases may be assessed not only if they present with early-onset of the disease or have a familial aggregation of breast and/or ovarian cancer. *BRCA1* and *BRCA2* mutation carriers are frequently diagnosed with triple-negative breast cancer [14]. Nevertheless, the German clinical selection criteria do not recommend *BRCA1* and *BRCA2* mutation screening in TNBC. In contrast, recent guidelines published by the National Comprehensive Cancer Network in the United States are less stringent. For instance, TNBC diagnosed before age 60, and breast cancer cases with a negative family history for cancer younger than 45 years should be tested according to these guidelines. In comparison, the German guidelines recommend testing single cases only if the diagnosis was made before age 35 [9], [10], [17].

In summary, we found a 20% germline mutation rate (6/30 cases) in *BRCA1* and *BRCA2* genes in patients diagnosed with TNBC from Southern Germany and unselected for age of disease onset and familial aggregation of this malignant disease. In contrast to recent reports, an unexpected and high mutation frequency was seen in *BRCA2* (5/30; 16.7%) suggesting that this gene may play an important role in the development of TNBC when mutated. Our data suggest that Southern German patients diagnosed with TNBC may qualify for *BRCA1* and *BRCA2* mutation screening. Larger patient cohorts have to be investigated to validate our findings.
